# “This is our life now. Our new normal”: A qualitative study of the unmet needs of carers of stroke survivors

**DOI:** 10.1371/journal.pone.0216682

**Published:** 2019-05-08

**Authors:** Alexandra M. J. Denham, Olivia Wynne, Amanda L. Baker, Neil J. Spratt, Alyna Turner, Parker Magin, Heidi Janssen, Coralie English, Madeleine Loh, Billie Bonevski

**Affiliations:** 1 School of Medicine and Public Health, Faculty of Health and Medicine, University of Newcastle, Callaghan, NSW, Australia; 2 Priority Research Centre for Stroke and Brain Injury, Hunter Medical Research Institute, New Lambton Heights, NSW, Australia; 3 School of Biomedical Sciences and Pharmacy, Faculty of Health and Medicine, University of Newcastle, Callaghan, NSW, Australia; 4 Department of Neurology, Hunter New England Local Health District, John Hunter Hospital, New Lambton Heights, NSW, Australia; 5 Deakin University, IMPACT Strategic Research Centre, School of Medicine, Barwon Health, Geelong, Victoria, Australia; 6 School of Health Sciences, Faculty of Health and Medicine, University of Newcastle, Callaghan, NSW, Australia; University of Melbourne, AUSTRALIA

## Abstract

Many stroke survivors require care from informal carers such as family members and friends who may experience adverse impacts. This study aimed to qualitatively explore the unmet needs of carers of stroke survivors, and their preferences for interventions and support services. We conducted 24 semi-structured, qualitative interviews with carers of stroke survivors from the Hunter region, Australia. Inductive thematic analysis was used in the context of a needs-led framework to identify key themes of their unmet needs. Key unmet needs identified by carers of stroke survivors in this study centred on four main themes: (1) social relationships and support; (2) adequacy of information; (3) taking care of oneself; and (4) accessing appropriate services. Carers of stroke survivors desired the development of services which provide connectivity to information, training, education and community support; and inclusion in a community with social relationships and other carers of stroke survivors. Ongoing unmet needs often result in adverse health and quality of life outcomes for carers of stroke survivors. Co-designed programs and resources for carers, particularly relating to unmet needs in social, information, self-care and service access domains are needed.

## Introduction

Stroke is often called a “family disease” with the potential to affect the health and quality of life not only of the person who has experienced the stroke, but also the family, friends and people who care for the survivor[1, 2]. Informal carers are “an unpaid individual, such as a family member, neighbour, friend or other significant individual, who takes on a caring role to support someone with a diminishing physical ability, a debilitating cognitive condition or a chronic life-limiting illness”[3]. Around one-half of people who experience a stroke event will require support from a carer[4], and the numbers of people providing informal care is expected to increase dramatically with demographic changes worldwide[5]. Meeting the needs of informal carers, safeguarding their health, and recognising the critical role they play have been identified as international priorities for support and policy changes around the world[5].

Unmet needs refer to a need that has not been satisfied yet. While some unmet needs and priority areas of needs have been identified in carers of stroke survivors, very few studies investigate these needs across settings and across difficult to reach populations, such as parents of young people who have had a stroke. In addition to the needs of carers of stroke survivors changing over time[6], their needs can also vary due to their age and time spent caring for the stroke survivor. For example, young carers of stroke survivors (under 60 years old) have reported feeling that services did not provide proper support for them due to their age-group[7]. Carers of stroke survivors have reported ongoing dissatisfaction with information provision and delivery[8–10], service accessibility[11–13], and struggling to cope with uncertainty[14], and the emotional, psychological and personality changes in the stroke survivor[15–17]. Further refining the unmet needs of carers of stroke survivors is needed to develop tailored resources and programs to meet these needs, and improve carers’ health and wellbeing.

Previous research has described the challenges of implementing carer interventions[18], including the need for most interventions to be tailored for not only the carer, but also the organisation delivering the intervention[18] and not addressing the ongoing complex and unique needs that change over time[18–20]. One way to overcome these challenges is to involve people from the group which the intervention aims to support, and engage with them to provide meaningful involvement in the development of acceptable and usable interventions in healthcare settings[21]. Engaging with carers of stroke survivors directly to identify their unmet needs, and in turn explore their preferences for resources to meet these needs provides a foundation for the development of acceptable programs. This engagement and consideration of the carers’ perspectives provides an opportunity for co-designed interventions, which in turn increases the likelihood that support and resources developed from this research will be utilised and adhered to.

Qualitative research can provide rich and unique insights into the experiences and unique needs of spouses, parents and adult children who provide care to someone who has experienced a stroke. As there has been an in-depth exploration of new carers of stroke survivors’ experiences and needs in the rehabilitation setting[22], this study seeks to expand this knowledge by identifying the unmet needs of carers of stroke survivors across a variety of settings and include the viewpoints of community and long-term carers. Furthermore, this research will provide directions for the development of acceptable and feasible programs/services which have been identified by carers of stroke survivors to meet their unique, complex and often ongoing needs. This study aims to qualitatively explore: (1) the unmet needs of carers of stroke survivors; and (2) their preferences for interventions and services to address these unmet needs.

## Methods

### Study design

Semi-structured phone interviews were conducted with carers of stroke survivors using a discussion guide and a brief survey of participant demographics. The discussion guide focussed on carers perceptions of their unmet needs, and preferences for services and interventions to address these unmet needs. Interviews were recorded with audiotapes and transcribed verbatim. The study design incorporated all aspects of the Consolidated Criteria for Reporting Qualitative Research (COREQ)[23] to ensure subsequent rigour in our reporting of our study.

### Setting

Participants were recruited from the Hunter Region, NSW Australia. The study received approval from the Hunter New England Human Research Ethics Committee Approval No. 17/09/20/5.07.

### Sample

Participants were eligible for this study if they were: (1) informal carers for someone who had a stroke; (2) 18 years or older; and (3) were able to participate in English. Formal carers, such as nurses, doctors and/or professional carers were excluded.

### Procedure

Two access points in the Hunter Region were used to approach potential participants and distribute study materials: the Community Stroke Service and the Hunter Stroke Research Volunteer Register. These organizations were chosen for recruitment as they were best suited to reach our target population. Participants were also recruited from support groups and by word of mouth. Stroke survivors who were registrants on the Hunter Stroke Research Volunteer Register were contacted by the staff by mail to ask someone, who they deemed to be a carer for them, to participate. Carers who wished to participate then provided written informed consent to the staff at the Hunter Stroke Research Volunteer Register who then passed the information to the research team. Participants could also contact the research team directly. Stroke survivors were contacted by the staff the Hunter Stroke Research Volunteer Register by mail and by follow-up telephone contact two weeks after the research packages had been sent. Participants then provided written informed consent to the Hunter Stroke Research Volunteer Register who then passed the information to the research team. Participants could also contact the research team directly. All interviews were conducted by one member of the research team (AMJD). Before the interview, participants were asked to complete a brief demographics survey. Following the survey, they were interviewed about their experiences and needs as a carer of a stroke survivor.

### Reflexivity

Reflective practice was part of the study, from the onset through to the analysis and interpretation. All interviews were conducted by one member of the research team (AMJD). AMJD was a female doctoral student and research assistant in the research area of health and medicine who had been previously awarded a Bachelor of Psychology (1^st^ class Honours). AMJD also had previous experience and interest in stroke-related research, in which she performed qualitative research during her Honours year of the Bachelor of Psychology, participated in NVivo training, and met with a qualitative expert independent to the work for specialised technique advice. Though no official relationship was established with participants before conducting the interviews, AMJD had had some contact with participants before they were interviewed. AMJD had attended the Community Stroke Service and support groups to promote the study, where she met some of the participants who were interested in the study. She also contacted participants to arrange a time which may be suitable to complete the interview. Due to the ethics approved information statement and study materials, participants were aware of AMJD’s study interests and reasons for conducting the research, though they were unaware of her personal background and/or motivations. During the research study, the interviewer and supervisor team reflected upon potential influence of the study on the interviewer, and to maintain an awareness of how the interviewer’s prior knowledge/experience could impact upon the work. Consequently, the ethics approved interview guide and prompts were adhered to conduct the interviews to assist in addressing potential interviewer bias.

### Measures

#### Demographic and caring characteristics survey

Participants were asked demographic information about themselves: age, gender, Indigenous status, marital status, employment status, and level of education. Carer-related information collected included length of the time the person has been a carer and their relationship to the stroke survivor. The age, gender and Indigenous status of the stroke survivor were also collected.

#### Discussion guide

The themes of the discussion guide were identified through literature review, and incorporated aspects of a needs-led framework[24] in which Socratic questioning[25] was used to explore the personal needs and experiences as a carer of a stroke survivor. The needs-led framework[24] was developed to provide a framework for conceptualizing and understanding how providing care impacts on carers’ fulfilment of needs. In this way, unmet needs were identified, and possible programs and services to meet these needs were discussed. The discussion guides were informant-led where possible, to allow issues and themes to arise from the interviews that were not part of the discussion guide (S1 Appendix). Broad prompts were available to the interviewer to explore key themes and experiences presented by the carer during the interview. Questions around carers’ preferences for services was introduced as a specific question theme if carers did not naturally provide information on this during the discussion. A protocol was developed to respond to participants who were feeling distressed, and a support document was available to direct participants to support such as Lifeline[26] and Carer Gateway[27].

### Data analysis

Demographic data were analysed descriptively. During each interview, AMJD recorded notes regarding points and themes of interest. After each interview, AMJD reflected on the emerging themes which then informed subsequent interviews. This iterative process was ongoing through the data collection period, and was used to assess data saturation when no new themes were emerging during interviews. Formal data analysis and content coding was performed after all interviews were completed and transcribed.

The content of interview transcriptions were analysed using NVivo 12[28], a software program designed for aiding thematic analysis of qualitative data. Key themes were independently coded by three reviewers, and discrepancies resolved by discussion and agreement. Two reviewers (AMJD and ML) independently coded all interviews; and a third reviewer (OW) coded seven (34.3%) interviews. The third reviewer (OW) was a female, more experienced post-doctoral researcher. Her different perspective provided further insight to the analysis of the transcripts and further development of the codes, which allowed the team to explore the richest possible interpretation of the data. An example of previous research where multiple coders were used to enhance qualitative analysis by providing different expertise insight is in Berends and Johnston[29]. The third reviewer was also referred to if disagreements occurred between the two reviewers AMJD and ML. Data were analysed using inductive thematic analysis[30]. The transcripts were analysed using Interpretative Phenomenological Analysis (IPA) [30–32]. The three stages of analysis included: (1) interviews are completed and transcribed verbatim; (2) reviewing and coding all patterns of information, themes and categories across interviews; and (3) identifying, comparing, and discussing themes with other reviewers. Quotes are presented to illustrate key themes; the relationship of the carer to the survivor and their participant identifier number follows each quote.

## Results

### Sample

Invitations to participate in the study from Hunter Stroke Research Volunteer Register were sent to 84 people with stroke to ask someone, who they deemed to be a carer for them, to participate. Of these, 12 (14.3%) participants identified as being recruited from the Hunter Stroke Research Volunteer Register. Three participants were recruited from the Community Stroke Team; and nine were recruited from other sources such as word of mouth and support groups. Sampling was ceased when saturation was met, and a maximum variation sample had been obtained.

The majority of participants were female (n = 19, 79.2%) spouses (n = 20, 83.3%) of the person who had experienced a stroke. Participants had cared for a person who had experienced a stroke for an average of 5.7 years, and ranged from three weeks to 17 years of providing care. The majority of all stroke survivors were male (n = 13, 54.2%) and ranged from 5–85 years old (M = 59.9, SD = 19.8). All stroke survivors identified as non-Indigenous (n = 24, 100%). Two of the three children who had experienced a stroke and were being provided informal care by their parent were under the age of 18 years (n = 2, 66.7%). Carer demographics are reported in Table 1.

**Table 1:** Carers of stroke survivors demographics, n = 24.

	Range	Mean (SD)
**Age in years**	30–87	56.83 (SD = 15.3)
**Time spent as a carer (days)**	21–6241	2080.9 (SD = 1809.2)
	**n**	**%**
**Gender**		
→Male	5	20.8
→Female	19	79.2
**Indigenous status**		
→No	24	100
**Marital status**		
→Married	20	83.3
→Defacto, or living with a partner	2	8.3
→Never married or single	2	8.3
**Education**		
→High school years 7–10	3	12.5
→High school years 11–12	4	16.7
→TAFE	7	29.2
→University	10	41.7
**Employment Status**		
→Full time or part time	12	50
→Retired	9	37.5
→Home maker	2	8.3
→Student	1	4.2
**Relationship with stroke survivor**		
→Partner/Spouse	16	66.7
→Adult child	4	16.7
→Parent	3	12.5
→Other family member (Aunt)	1	4.2
**Live with stroke survivor**		
→Yes	20	83.3
→No	4	16.7

### Themes

Interviews with participants averaged 42 minutes and ranged from 22 minutes to 1 hour.

Analysis identified four key themes: (1) social relationships and support; (2) adequacy of information; (3) taking care of oneself; and (4) accessing appropriate services. Each need and how it impacts the carers’ lifestyle is described below. Table 2 shows representative quotes from each theme and category.

**Table 2:** Unmet needs reported by carers of stroke survivors, and representative quotes from each domain.

Domain	Representative Quotes
**Social relationships and support**	
	*“My wife’s family …dropped off the Earth*. *We haven’t seen them since the stroke and I mean that was hard*, *that was part of [my wife’s] depression*.*”* **(Male Spouse, Participant 16)** *“I guess it's really important to find some sort of other carers that are in similar situations*. *Because your friends they don’t get it*. *…Even*, *some of my other carer friends*, *their partners don’t have aphasia*, *they don’t get it like my friends from aphasia group get it*.*”* **(Female Spouse, Participant 13)** *“I feel a bit of a minority because there are not as many kids that are stroke affected there … there’s nothing specifically for kids with stroke so that’s what I found is the problem*.*”* **(Mother, Participant 2)** *“They always hold them during weekdays*, *during work hours when I’m never able to attend*. *That makes it so hard because the only time I have free is the weekends*.*”* **(Female Spouse, Participant 18)** *“I'm already trying to fit everything else in for everyone else and*, *do I have to fit in a psychologist or a counsellor too*, *do I*?*”* **(Female Spouse, Participant 13)**
**Obtain adequate information**	
	*“I would say that the first part of [my husband]’s experience at the hospital and my experience at the hospital was horrendous … They were in the middle of a wages dispute*,*… He wasn’t showered for the first five weeks that he was in the hospital*. *It was awful*, *and the staff would keep saying “[my husband] won’t remember any of this*.*” No he won’t*, *…*. *But I'll never forget it*.*”* **(Female Spouse, Participant 12)** “*You don't have to agree with them (doctors) 100% of the time*. *[after having our concerns dismissed]*, *we went to another doctor and literally that day [he] sent one referral onto the paediatric unit at the local hospital*.*… If I had left it*, *it would have been later that she entered the intervention programme and she may not have been as mobile and as talkative as she is today*.*”* (**Mother, Participant 2)** *I was given a pack from the hospital and that’s it—I had to take home and read*. *I was terrified*. *There was just no one for me to talk to*.*”* **(Female Spouse, Participant 18)** *I didn’t know what to expect and I wasn’t given any directions or any assistance or anything you know*. *So initially it was just frightening*.*”* **(Female Spouse, Participant 1)**
**Take care of oneself**	
	*“I feel I’ve become a little bit introverted during this process and whereas I might’ve met a friend for coffee or lunch or whatever*. *I just don’t seem to get the space to do that now*.*”* **(Female Spouse, Participant 5)** *I’m so stressed out my ulcerative colitis has flared up*, *I’m doubled over in pain most of the day at work*. *I just never get a break*. *I’m just miserable to be honest*.*”* **(Female Spouse, Participant 18)** *There could have been more attention paid to the carer*. *In saying*, *this is what’s going to happen*, *or this is what’s happened*, *this is what your wife has experienced*, *this is how you manage it*.*”* **(Male Spouse, Participant 17)**
**Service accessibility**	
	*“We basically left that hospital with no information at all*.. *… When you’re talking to them they say “Oh have you done this*? *Have you done that*?*” No we haven’t*, *no-one told us about that*.*”* **(Male Spouse, Participant 16)** *“In some ways gets harder and that’s part of our dilemma that the support we received initially*, *can’t continue and you start to feel a little bit isolated … you’re left*, *it’s assumed well you know what to do now”* **(Female Spouse, Participant 5)**

#### Unmet needs theme one: Social relationships and support

Participants often described how their social support networks with family and friends changed as a result of caring. Spouses in particular expressed changes in relationship dynamics and/or feelings of abandonment by friends or intimate support networks. Most participants also described how providing care for the person who had a stroke impacted on other relationships, reduced their social networks and often resulted in isolation following the stroke event. Other participants described that their loss of support networks may have been due to self-preservation, “W*e’ve probably isolated ourselves a little bit as well just to survive*.*”* **(Female Spouse, Participant 5).**

Additionally, participants often expressed the importance of connecting with other people who care for a stroke survivor. Participants reported valuing interactions with other carers; sometimes more so than interactions with family and friends. Spouses in particular described the importance of seeking other carers of stroke survivors to share their experiences with, “*Other people that get what you're going through*, *not just can sympathise or empathise… but really get it because they’re living it too*.*”* **(Female Spouse, Participant 12).**

Participants described various barriers to accessing social support available in the community, such as support groups and networks. The biggest challenge identified by the vast majority of participants to accessing community support was managing time commitments. Other barriers which restricted the participants’ ability to attend and access available community support included anxiety around separation from the stroke survivor to attend support groups, receiving age-appropriate support in the community, and providing care for other dependents such as children.

Further sources of support were health care practitioners (especially doctors) and the health care system. Participants’ experiences and unmet needs in this area were mainly related to communication and will be presented within the theme related to ‘obtaining information’.

#### Unmet needs theme two: Obtain adequate information

The need to obtain adequate information was prevalent across the majority of the participants’ experiences. Most participants distinctly recalled the first interaction with the health care team with vivid clarity from the time that the person had a stroke. Most participants discussed the need to receive support from health care professionals across settings, particularly relating to inadequate information and resources about the caregiving role. Negative experiences with health care and service providers related to poor communication with the carer and deliberately ignoring carer concerns.

Participants discussed feeling dissatisfied and disappointed with information provision and delivery, and discussed improvements on how they received information. Interestingly, new carers described experiencing similar dissatisfaction as long-term carers. Participants also expressed dissatisfaction with the type of information delivered, for example some received booklets and leaflets in a package which they never referred to. Participants also recalled the timing of the information was often delivered when they were feeling completely overwhelmed. In addition to the type of information and when the participants’ received the information, they recalled the person, or lack thereof, who would deliver the information. Additionally, it was often mentioned that even if they had the opportunity to approach healthcare staff, the participants’ didn’t know what to ask. Overall, there was a sense that many participants were left without guidance, and unprepared for the caregiving role. “*I didn’t know what to expect and I wasn’t given any directions or any assistance or anything you know*. *So initially it was just frightening*.*”* **(Female Spouse, Participant 1).**

#### Unmet needs theme three: Take care of oneself

Participants often discussed the impact of caregiving on their autonomy, mental and physical health and wellbeing. Participants often described that their free time was available in limited windows. For example, *“… the only time I get a bit of free time is if she goes to bed*.*” (***Male Spouse, Participant 4**), or is being looked after by another person, such as a formal carer for a few hours. Participants were often aware that their health was declining, but they couldn’t find the time to balance their own needs in addition to those of the people that they cared for. *“The only advice I got was you’ve got to look after yourself*. *Well*, *how do you do that*?*”* **(Female Spouse, Participant 1).**

The majority of participants expressed difficulty in separating and managing their own needs from their role as a carer. Some carers discussed dealing with poor management of their physical and mental health in addition to providing care. Some participants had more general comments and observations about how their mental and physical self-care had diminished, *“I focused on my work*, *my family and my husband to the physical detriment of myself*.*”* **(Female Spouse, Participant 15)** but often the physical and mental health issues manifested themselves together, and participants did identify the cause of their health decline to be providing care.

One of the biggest challenges that participants faced was emotional distress and feelings of grieving for their lost lives, identity and role changes. This included aspects of carers’ uncertainty about events that are inherently unpredictable—including the mental and physical changes in the stroke survivor, the risk of having a second stroke or level of functioning. Working-age spouses in particular were devastated by their *“new normal”* and uncertainty about the recovery of their spouse who had experienced the stroke. *“I thought that there’ll be a gradual return to work you know going back to life as it was and I think that’s*, *that’s a lot of the grieving that we both experience that this is*, *this is our life now*.*”* **(Female Spouse, Participant 5).** Personality changes were often one of the most difficult mental and physical adjustments for carers. “*He’s a completely different person*, *I call him [husband] version two*.*”* **(Female Spouse, Participant 18)**.

#### Unmet needs theme four: Service accessibility

Difficulties identifying and finding appropriate services were common. However, issues still remained once carers had identified a service. For example, some participants reported that the National Disability Insurance Scheme (NDIS)[33], an Australian insurance scheme which provides support for people with disability, their families and carers, was a helpful service for those who could access it, and was acknowledged by carers that it was a positive resource available to new carers for receiving supporting and navigating the healthcare system. However, many also felt frustrated by the NDIS due to restricted availability of financial support and lack of ongoing support options. “*At first the NDIS provided lovely care*. *Every week day*, *and a couple of hours on the weekend*. *On a Saturday*. *But this year the support has just disappeared*.” **(Mother, Participant 23).**

New carers described difficulty in making arrangements when the person that they cared for was discharged *“I keep asking what will happen when he comes home*? *What services will be in place for him and I*? *And what I’m quickly discovering is that they’ll wait for a disaster and then they’ll deal with that*.*”* **(Female Spouse, Participant 14).** Some participants talked about how they utilised health insurance and income protection, but it was often difficult to access during the urgent time immediately after the discharge home. Participants described further out-of-pocket costs for services such as home modifications, but most justified the expenses as an improvement to the health and wellbeing of the stroke survivor. *“It’s still a financial drain but we will try anything that will improve quality of life*.*”* (**Female Spouse, Participant 15).**

Generally participants who had been providing care for a longer period of time recalled their experiences in trying to arrange services to meet their initial needs. *“I was unable to get any help*, *government help because most of the companies that supply their home care*, *their books were full*. *When I needed the help the most*, *in the first three months I was unable to get it*.*” (***Female Spouse, Participant 10).** Furthermore, carers who had been providing care for longer periods of time identified the lack of support for ongoing services to meet their needs.

### Preferences for services and interventions

Two broad categories of types of preferred services and interventions were identified: (1) Connecting carers to a single service that provided ongoing information, education, services and support; and (2) Inclusion in a community and social activities with other carers.

Carers for the most part wanted access to an ongoing service which they could either contact themselves or which regularly checked in with them over time to receive support and guidance. Examples included someone to guide them to support and information, *“So [they call a week later and say*: *“How are you going*?*” “You know*, *I need help for showering or something*,*” and then they would say*: *“Well try this*,*”* **(Female Spouse, Participant 10)** or available access to someone who could help them as new issues arise, “*I have had moments in regards to Dad where*, *he's sort of been*, *behaving in a certain way and I've just thought*, *I wish there was a number I could call”* **(Daughter, Participant 9).** Most carers felt that an ongoing service would be beneficial in assisting them with providing care and meet the ongoing complex and often changing needs of being a carer to a stroke survivor.

Some of the participants had investigated online platforms as a way to connect with other carers. However, in some cases these were also described as being less than adequate and often not suited to Australian carers, *“I’m a member of a paediatric stroke page overseas*. *They do have a small one that is based in Australia but not as well organised*.*”* **(Mother, Participant 2).** Some carers identified that the services and support they desired were available to other groups of people with disability “*You know*, *[support] is readily available for many other diseases and illnesses*, *like cancer and breast cancer*. *The breast cancer people have done a wonderful job in providing support to survivors of breast cancer*. *You know*, *there’s stuff available there*, *but… nothing is promoted for stroke*.*”* **(Male Spouse, Participant 17).** As a result of needing better services to address their own needs, and the needs of the people that they cared for met, some carers turned to advocacy for their loved ones. “*I learnt very quickly that you had to be kind of like as a carer … you had to be an advocate for your loved one and you had to stand up and you had to speak until you were heard …”* **(Female Spouse, Participant 15).**

Most carers also desired inclusion in a community with other carers of stroke survivors. Types of community were discussed: some carers desired to connect with other carers face-to-face to learn about others’ experiences, and peer-to-peer mentoring within the carer community. Carers also discussed engaging in online groups with other carers. The majority of carers desired to meet people in a relatable situation to receive and provide support across a variety of platforms.

## Discussion

Inductive thematic analysis was used in this study to capture the unmet needs of carers of stroke survivors, and their preferences for support services and interventions. Participants discussed their experiences as an informal carer of a stroke survivor, and from this, key themes of unmet needs were identified. Carers identified their main preferences for services and interventions were those which met their ongoing needs, and allowed them to engage with a community of people who were also carers of stroke survivors.

Many carers expressed feeling isolated and alone due to the deterioration and/or lack of quality of their social relationships and support following the stroke event. Carers described how their relationships with friends and family, health care professionals and community support were impacted post-stroke. Our findings were consistent with previous research, and found that these are the main needs that are still unmet: friends of the carer and stroke survivor may avoid contact because stroke survivors have problems communicating, mood changes, or a reduced interest in socialising[34, 35]; feelings of isolation, abandonment and lack of support from family members and friends[17]; dissatisfied with their interactions with health care professionals and the type of information received during the rehabilitation and discharge period[22] and lack of ongoing support[6, 36, 37]. Furthermore, most participants discussed a decline in their mental and physical health as a result of providing care, and few had strategies for managing their health, or knew how to access services if they were available[5, 38, 39]. Providing carers of stroke survivors’ access to ongoing supportive services such as counselling support, financial assistance and general health advice may improve their health and wellbeing outcomes.

A novel contribution of this study is the exploration of carers’ first-hand testimonials of their preferences for support, which provides a strong basis to develop resources which have been co-designed by carers of stroke survivors. Future research is needed to develop and refine evidence-based support services and resources that carers of stroke survivors desire to assist them with their ongoing needs and increase their health and well-being. Interventions, support services and programs which consider appropriate platforms and different demographics which have been developed in collaboration with carers of stroke survivors have the potential to improve health settings, as more resources and education for carers of stroke survivors may be integrated into routine stroke care.

### Implications for policy and practice

Carers’ preferences for services and interventions to meet their needs were largely the need for development and services available to provide connectivity, inclusion, and ongoing care. For example, one requested feature was a hotline where a carer of a stroke survivor could contact someone for support and advice. This service exists in Australia for carers of people who have cancer, provided through resources developed by the Cancer Council[40], which provides ongoing up-to-date information and support relating to: (1) online support; (2) in person and over the phone support; (3) practical support services; and (4) information for carers[40]. In order for stroke survivors to receive a service like this, there is a need for strong advocacy for consumers (both stroke survivors and their carers) and families. These findings highlight the need for government bodies and non-government organisation charities to recruit supporters who are staff, volunteers, and stakeholders to commit and prioritise reducing the impact of stroke on individuals, their carers, families, and the community.

### Study limitations

A limitation of this study is that participants were recruited from a metropolitan area, potentially restricting the transferability of findings to the needs of carers of stroke survivors in other areas, such as rural areas, who may experience different and unique challenges. Another limitation is that the study included a heterogeneous sample of carers with respect to age and relationship to the survivors. While heterogeneity has value in the early stages of investigation of a field, it restricts the richness of information pertinent to subgroups. For future research, particular subgroups such as parents of stroke survivors may be targeted to provide additional perspectives to the role of providing care for a stroke survivor.

The incidence of stroke among young people is rising[41], and as a result, the needs and experiences of younger informal carers are becoming an increasing priority for exploration and support. The needs of parents who provided care for a child who had a stroke were broadly very similar to the needs of working-age caregivers and stroke survivors. Only three parents were carers of children who had stroke and the results shared similarities with other caregiver groups. For example, both parent and non-parent caregivers struggled to find support for people in similar situations to themselves, age-appropriate support for the person that they cared for, a need for clearer communication and support from health care providers on the prognosis of the younger stroke survivor, and uncertainty for the future around the recovery of the stroke survivor as a younger person. In this study, there was little difference found in the experiences of carers of children and other carers. Further research should focus on the exploration of these hard-to-reach carer subgroups and explore their unique needs and challenges as a carer.

## Conclusions

Carers of stroke survivors experience a range of needs which are not supported by current available supports and services. Carers of stroke survivors desired the development of services and support which provided connectivity to information, education and community support. This study provides a foundation for the development of resources, programs and interventions which are co-designed by carers of stroke survivors and can provide carers with information and support to meet their unmet needs.

## Supporting information

S1 Appendix Discussion guide. The discussion guide used during the study to interview participants.
(DOCX)
