# Supplementary material for: “This is our life now. Our new normal”: A qualitative study of the unmet needs of carers of stroke survivors
Source: PLoS One. 2019 May 8;14(5):e0216682. doi: 10.1371/journal.pone.0216682 (PMC6505885; doi:10.1371/journal.pone.0216682)
Supplement: S1 Appendix — The discussion guide used during the study to interview participants. (DOCX) [file pone.0216682.s001.docx]

**S1 Appendix. Discussion Guide.**

**The Unmet Needs of Carers of Stroke Survivors: A Qualitative Study *Discussion Guide***

Hi, my name is xxx. I am a researcher at the University of Newcastle.

You have been invited today to talk about your personal needs and experiences as a carer of a stroke survivor. I am also interested on your thoughts about programs and services that may help you.

The interview will take approximately 30-45 minutes. Although I’ll be asking some specific questions to guide our chat today, we can also explore any other aspects of your caregiving experience that you would like to discuss today.

To begin I would like to go over some aspects of the Information statement. Firstly, anything that you say during this chat will remain anonymous. Our conversation today will be audio-taped however no names or identifiers will be used during reporting. If you wish to have any of your comments deleted we can do so at the end of the interview otherwise if you wish to no longer participate at any stage then just let me know and we can stop.

Do you have any questions that you would like to ask me?

1. **Introduction**

The first thing we will do is the brief survey. I will ask you some questions such as about yourself your relationship with the person that you care for. Are you ready to start the survey? Thank you – we will now begin the interview.

1. **Caring/Life course**

- What brought you to today? Tell me a bit about yourself.
- What led you to being a carer?
- What do you think of “caring” as?

1. **Typical day of providing care for a stroke survivor/needs**

- Tell me about your typical day of providing care for the person that you care for?
- What do you need during your day to provide care for [person that you care for]?
- Have these needs changed over time? Do you expect them to change more?
- Is the help out there “helpful”?

1. **Support**

- Do you receive much support from your family?
- What about support from healthcare providers, such as doctors and nurses?
- Has this support changed over time? Do you expect things to change more?

1. **Support and services that you think would help you.**

- What kinds of programs and services do you think would be most useful to you as a carer of a stroke survivor?
- (Prompt) Explore further – it sounds like xxx has been a problem for you. What do you think would help you manage xxx?

**Conclusion**

Thank you for speaking to me today. I would like to express how appreciative we are of you sharing your thoughts and experiences. Is there anything you would like to add?

Thank you again for all your comments.
